# Supplementary material for: Conditional chemoconnectomics (cCCTomics) as a strategy for efficient and conditional targeting of chemical transmission
Source: eLife. 2024 Apr 30;12:RP91927. doi: 10.7554/eLife.91927 (PMC11060718; doi:10.7554/eLife.91927)
Supplement: Figure 3—source data 3. [file elife-91927-fig3-data3.zip › Figure 3 source data3 part 1.zip!/Figure 3-Source data 3-part1/1063f3-1-CRISPResso_Report_kboyr13q.zip!/CRISPResso_on_1063f3_1/9.Alleles_frequency_table_around_sgRNA_aatcacaccagtgggagcac.pdf]

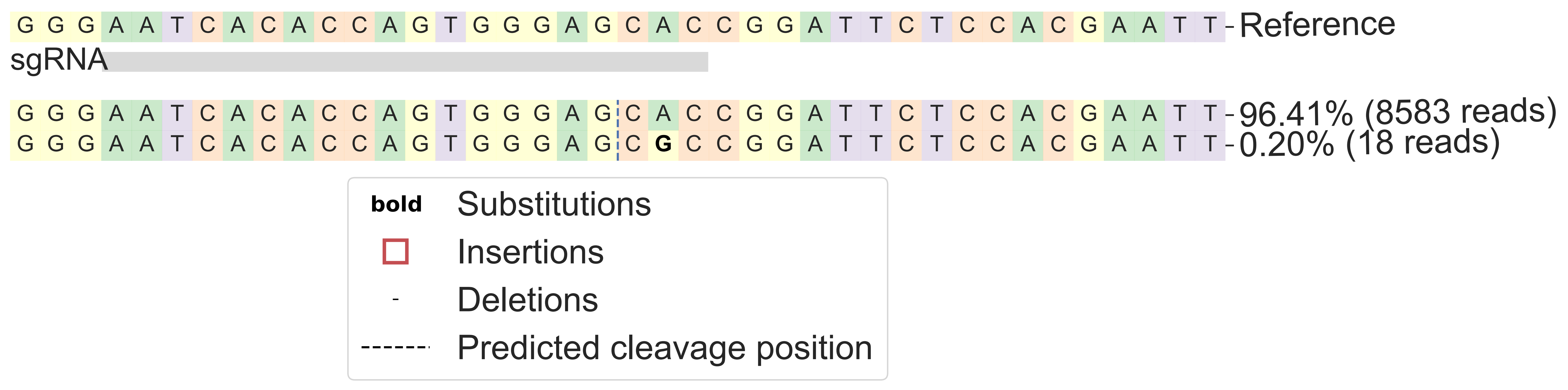
G G G A A T C A C A C C A G T G G G A G C A C C G G A T T C T C C A C G A A T T
Reference
sgRNA
G G G A A T C A C A C C A G T G G G A G C A C C G G A T T C T C C A C G A A T T
96.41% (8583 reads)
G G G A A T C A C A C C A G T G G G A G C G C C G G A T T C T C C A C G A A T T
0.20% (18 reads)
bold
Substitutions
Insertions
Deletions
Predicted cleavage position
